# Aging and Masculinity in Bibliotherapy and Poetry Groups for Men in Late Life

**DOI:** 10.1093/geroni/igab046.2172

**Published:** 2021-12-17

**Authors:** Dovrat Harel

**Affiliations:** Tel Hai College, Kidron, HaMerkaz, Israel

## Abstract

The transition to residential care facility may symbolize the joining into the 'community of older people'. This may influence the ways men in late life construct their identities and the intersection of aging and masculinity. Due to some barriers, this experience may be difficult to express explicitly. Bibliotherapy aims to bridge this gap by using literature to address diverse issues in the form of reading and writing activities. In this presentation we will present "The Literary Parliament" project, in which bibliotherapy and poetry groups of men in late life were conducted in residential care facilities in Israel. We will present findings of a qualitative research which explored poems written by group participants, and the way these helped participants to express their perceptions and feelings of ageing and masculinity in the context of residential care. The use of bibliotherapy to encourage psychological discourse among men in late life will be discussed.

